# The role of pathological tau in synaptic dysfunction in Alzheimer’s diseases

**DOI:** 10.1186/s40035-021-00270-1

**Published:** 2021-11-10

**Authors:** Moxin Wu, Manqing Zhang, Xiaoping Yin, Kai Chen, Zhijian Hu, Qin Zhou, Xianming Cao, Zhiying Chen, Dan Liu

**Affiliations:** 1 Department of Medical Laboratory, Affiliated Hospital of Jiujiang University, Jiujiang, 332000 China; 2 Jiujiang Clinical Precision Medicine Research Center, Jiujiang, 332000 China; 3 Medical College of Jiujiang University, Jiujiang, 332000 China; 4 Department of Neurology, Affiliated Hospital of Jiujiang University, Jiujiang, 332000 China; 5 Department of Dermatology, Wuhan No. 1 Hospital, Tongji Medical College, Huazhong University of Science and Technology, Wuhan, 430022 China; 6 Department of Medical Genetics, Tongji Medical College, Huazhong University of Science and Technology, Wuhan, 430030 China

**Keywords:** Pathological tau, Synaptic dysfunction, Synaptic plasticity, Alzheimer’s disease

## Abstract

Alzheimer’s disease (AD) is a neurodegenerative disease characterized by progressive cognitive decline, accompanied by amyloid-β (Aβ) overload and hyperphosphorylated tau accumulation in the brain. Synaptic dysfunction, an important pathological hallmark in AD, is recognized as the main cause of the cognitive impairments. Accumulating evidence suggests that synaptic dysfunction could be an early pathological event in AD. Pathological tau, which is detached from axonal microtubules and mislocalized into pre- and postsynaptic neuronal compartments, is suggested to induce synaptic dysfunction in several ways, including reducing mobility and release of presynaptic vesicles, decreasing glutamatergic receptors, impairing the maturation of dendritic spines at postsynaptic terminals, disrupting mitochondrial transport and function in synapses, and promoting the phagocytosis of synapses by microglia. Here, we review the current understanding of how pathological tau mediates synaptic dysfunction and contributes to cognitive decline in AD. We propose that elucidating the mechanism by which pathological tau impairs synaptic function is essential for exploring novel therapeutic strategies for AD.

## Introduction

Alzheimer’s disease (AD) is the most common neurodegenerative disorder, which potentially affects 50 million individuals worldwide and represents 60%–80% of dementia cases [1]. The pathological hallmarks of AD include extracellular amyloid-β (Aβ) plaques and intracellular neurofibrillary tangles (NFTs) in the brain, as well as loss of neurons and synapses, resulting in cognitive impairment and eventually dementia [2]. The pathophysiology of AD follows a stereotypical spatiotemporal pattern: Aβ plaques first appear in the frontal lobes, even before any clinical symptoms, followed by the appearance of NFTs in the entorhinal cortex and hippocampus, triggering a series of toxic events and ultimately leading to cognitive decline [3]. It is becoming clear that synaptic loss and synaptic dysfunction are two key components of the neurodegenerative process of AD, especially in early AD [4–6]. Although Aβ plaques precede NFTs in patients with AD, epidemiological studies suggest that the regional distribution of NFTs is highly correlated with the severity of cognitive deficits [2, 7]. As the main component of NFTs, tau is a microtubule-associated protein that has been identified as a key molecule in AD and a series of neurodegenerative diseases collectively referred to as tauopathies [8]. The pathogenic role of tau in neurodegenerative disease has been confirmed by the identification of *MAPT* mutations and polymorphisms in patients with frontotemporal dementia with parkinsonism-17 [9].

Synaptic dysfunction is considered to be an early pathological manifestation and key component of the neurodegenerative process of AD. Epidemiological studies have shown that synaptic loss is closely correlated with cognitive decline in patients with AD, suggesting that synaptic integrity plays a causal role in the etiology of AD [2, 10]. Importantly, studies in mouse models support that the accumulation of pathological tau triggers synaptic loss and deregulation without widespread neuron loss [11, 12]. In addition, growing evidence suggests that soluble forms of tau species, especially oligomers, rather than NFTs, are toxic to synapses [13–15].

Tau is predominantly present in the axonal compartments of neurons and its main function is to regulate microtubule assembly and stabilization, and axonal transport [13]. Some studies have shown that tau can also be detected in the dendrites and the pre- and postsynaptic terminals of normal healthy neurons, and participates in important physiological functions related to synapses [14, 16, 17]. Under pathological conditions, mutations or post-translational modifications of tau are able to lower its affinity to microtubules [13], leading to its detachment from axonal microtubules, and subsequent aberrant aggregation, missorting to subcellular compartments, and propagation to other brain regions [5, 14]. Pathological tau is produced from aberrant post-translational modifications, including phosphorylation, acetylation, ubiquitination and truncation, leading to conformational changes, aggregation, NFT formation and synaptic dysfunction in AD [18]. The pathological tau induces synaptic dysfunction in several ways, including reducing the mobility and release of presynaptic vesicles, decreasing glutamatergic receptors, impairing the maturation of dendritic spines at postsynaptic terminals, disrupting mitochondrial transport and function in synapses, and promoting the phagocytosis of synapses by microglia. Reducing endogenous pathological tau can protect against excitotoxicity and neuronal dysfunction, and ameliorate cognitive deficits in mouse models of familial AD and related conditions [19]. Elucidating potential pre- and postsynaptic pathways of pathological tau will help reveal the pathogenesis of synaptic dysfunction. Therefore, it is essential to better understand the relationship between pathological tau and synaptic dysfunction in AD, which will help to clarify the molecular mechanisms of tau-mediated cognitive dysfunction and provide constructive strategies for protecting synapses. Here, we review the recent research advances on the role of pathological tau in synaptic dysfunction and cognitive decline in AD.

## Overview of tau protein

Tau, a major microtubule-assembly protein, is encoded by the *MAPT* gene which comprises 16 exons, located on chromosome 17q21. In the adult brain, tau is mainly present in neurons, and at low levels in oligodendrocytes and astrocytes [20, 21]. The functions of tau in oligodendrocytes and astrocytes include transporting glutamate and maintaining the integrity of the myelin sheath and the blood–brain barrier [22–24]. Tau has long been considered as an axon-associated protein, as it binds to axonal microtubules and participates in microtubule stabilization and axonal transport [25]. The primary sequence of tau consists of four functional regions: the N-terminal region, a proline-rich domain, microtubule-binding repeats (MTRs), and the C-terminal projection domain [26]. There are six isoforms of tau in the mature human brain, including those having four microtubule-binding repeats (4R) with no amino-terminal inserts (0N), 4R1N, 4R2N, 3R0N, 3R1N and 3R2N, which are derived from alternative splicing at exons 2, 3 and 10 of the pre-mRNA [27, 28]. The different isoforms are present in distinct subcellular localization patterns in wild-type mice: the 0N isoform is highly expressed in cell bodies and axons, the 1N isoform is strongly localized to the neuronal nucleus, detectable in cell bodies and dendrites, but absent from axons; the 2N isoform is strongly localized in axons and cell bodies, and detectable in dendrites [29]. The different distributions of tau isoforms may reflect distinct physiological functions. Additionally, the 3R and 4R isoforms have a ratio of about 1:1 in the healthy human brain, and this ratio varies in neurodegenerative diseases [30]. As a substrate of a large number of kinases, tau potentially has 80 serine/threonine and 5 tyrosine phosphorylation sites which mainly cluster in the proline-rich region and the tail domain adjacent to the MTRs [27, 31]. The ability of tau to assemble microtubules is inversely related to its degree of phosphorylation [32]. Changes in the local microenvironment and insufficiency or degradation of molecular chaperones make tau prone to form paired helical filaments and ultimately lead to NFTs [33]. Although tau was initially described as an axonal protein, it can be mislocalized into dendrites and pre- and postsynaptic compartments in certain pathological contexts, and is involved in the blockade of neuronal signaling and impairment of synaptic plasticity [34–36].

## Physiological roles of tau in synaptic plasticity: lessons from tau knockout mice

Synaptic plasticity refers to the activity-dependent enhancing or weakening of the existing synapses, including changes in the number of synapses, morphological structures, and function. Synaptic plasticity is crucial to cognitive functions, including learning and memory [37]. Impairments in synaptic plasticity cause defects in synaptic function, ultimately leading to neurodegeneration and loss of neuronal connectivity [38]. The translocation of endogenous tau from dendrites to excitatory postsynaptic compartments induced by pharmacological stimuli implicates that tau may be involved in the regulation of synaptic plasticity [39]. Some studies have reported that tau depletion does not impair cognition [19, 40], while others have observed synaptic plasticity and memory deficits in tau knockout (KO) mice. Chen et al. [41] have revealed that tau is involved in the regulation of the brain-derived neurotrophic factor-induced morphological synaptic plasticity in hippocampal neurons. Tau plays a critical role in certain cognitive functions and tau KO mice demonstrate impairment of long-term potentiation (LTP), which is a form of synaptic plasticity that is regarded as the primary cellular substrate for learning and memory [42]. On the other hand, Kimura et al. [43] have found a selective defect of long-term depression (LTD) in tau KO mice, and this effect can be replicated through knocking down tau by RNA interference in hippocampal slices of the CA1 region. As a weakening of synaptic strength following a stimulus, LTD is thought to be fundamental for clearing old memory traces [44]. Phosphorylated tau, mediated by glycogen synthase kinase 3 (GSK-3β), is required for *N*-methyl-*D*-aspartic acid receptor (NMDAR)-dependent LTD and has a critical physiological function in promoting LTD [43]. Regan et al. [45] have also observed impaired internalization of α-amino-3-hydroxy-5-methyl-4-isoxazolepropionic acid receptors (AMPARs) in the hippocampus of tau KO mice, and that tau phosphorylated specifically at serine 396 and 404 residues can promote LTD. Additionally, tau KO mice show reductions of the insulin-induced LTD, which is caused by altered activities of the tumor suppressor PTEN (phosphatase and tensin homologue on chromosome 10) [46]. A recent study has shown that *Mapt*−/−mice with a distinct genetic background display synaptic plasticity defects, hyperactivity and aging-dependent short-term memory impairments, while partial deletion of tau (*Mapt*+/−) only shows a mild short memory deficit [47]. The results on cognitive impairments may depend on the genetic background of mice, the use of different *Mapt*−/− models, the type and conditions of memory tests performed, and variations in housing and diet conditions. Therefore, it is important to further explore the role of tau in synaptic plasticity in the context of aging, which could further shed light on why certain forms of tau cause neurotoxicity in mature and/or aging brains.

## Synaptic dysfunction in AD

In the central nervous system (CNS), synapses are considered to be the fundamental units for transmitting electrical or chemical signals among neurons, and are subject to strict spatiotemporal regulation [10]. Synapses are composed of three specialized compartments: the presynaptic terminal which is dedicated to the coordinated release of vesicles containing neurotransmitters [48]; the postsynaptic compartment which transduces the incoming signals through neurotransmitter receptors such as AMPARs and NMDARs [49]; and the synaptic cleft which is located between the pre- and postsynapse. Postsynaptic density (PSD) is an assembly in the postsynaptic compartment, and refers to the postsynaptic receptor-rich portion that harbors a series of scaffolding proteins and metabotropic receptors [50]. Moreover, other neural cell types such as microglia can contribute to synaptic maintenance and function [51].

Synaptic dysfunction may lead to some forms of neurodegeneration and dementia, highlighting the importance of synapses for healthy brain functions [52]. Patients with AD have fewer synapses than healthy controls [53]. Synaptic loss is described as an early indicator of neuronal dysfunction and the best biologically relevant factor for disease progression of AD [2, 54, 55] and other tauopathies [56–58] such as frontotemporal dementia, progressive supranuclear palsy, corticobasal degeneration and Pick’s disease. Synaptic dysfunction includes changes in the morphology and function of presynapses, dendrites, postsynapses, and synaptic clefts. Mitochondrial energy supply disorder and phagocytosis of synapses by microglia may also contribute to synaptic dysfunction.

## Pathological tau and synaptic dysfunction

It is generally considered that tau is absent in dendrites, except for developing neurons or when under pathological conditions. However, extensive emerging evidence suggests that tau can also be detected in dendrites and pre- and postsynaptic compartments of normal healthy neurons, and is involved in the regulation of synaptic function [11, 14, 28]. In healthy human brains, tau is detected in 55% of presynaptic compartments and 70% of postsynaptic compartments [9]. The presence of tau in dendritic and synaptic compartments opens up a new and convincing chapter on the function of tau. However, its physiological and pathological effects are still not well-understood, especially in terms of learning, memory, and cognition.

Synaptic dysfunction is an early pathological feature associated with pathological tau in AD. During the pathogenesis of AD, the mechanisms involved in synaptic plasticity are dysregulated, leading to synaptic dysfunction and collapse [5, 59]. Pathological tau in sporadic AD has reduced microtubule-binding affinity, resulting in its detachment from axonal microtubules and subsequent mislocalization into somatodendritic compartments, impairing the integrity of microtubules and inducing synaptic dysfunction [5, 11, 31]. In fresh post-mortem tissues of patients with AD, phosphorylated tau has a higher level in the hippocampus and entorhinal cortex than in the neocortical regions, and accumulates at synaptic terminals [17]. The accumulation of pathological tau in the synapses can promote synaptic loss and synaptic plasticity impairment [5, 60–62]. The density of NFTs is strongly correlated with synaptic loss and cognitive decline, suggesting that the pathological tau may be a pathogenic factor [63]. Reducing the levels of endogenous tau can prevent synaptic dysfunction in a mouse model of AD, which is mediated by changes in postsynaptic molecules [34, 64]. Additionally, synaptic defects are also associated with the soluble form of tau preceding the NFT formation, which implies that the soluble tau is sufficient to drive synaptic dysfunction [65, 66]. AD patients with relatively high levels of pathological tau have more severely impaired synaptic plasticity and faster cognitive decline [67]. However, the effect of pathological tau on synaptic dysfunction is still not fully understood.

### Pathological tau induces presynaptic dysfunction

**Fig. 1:**
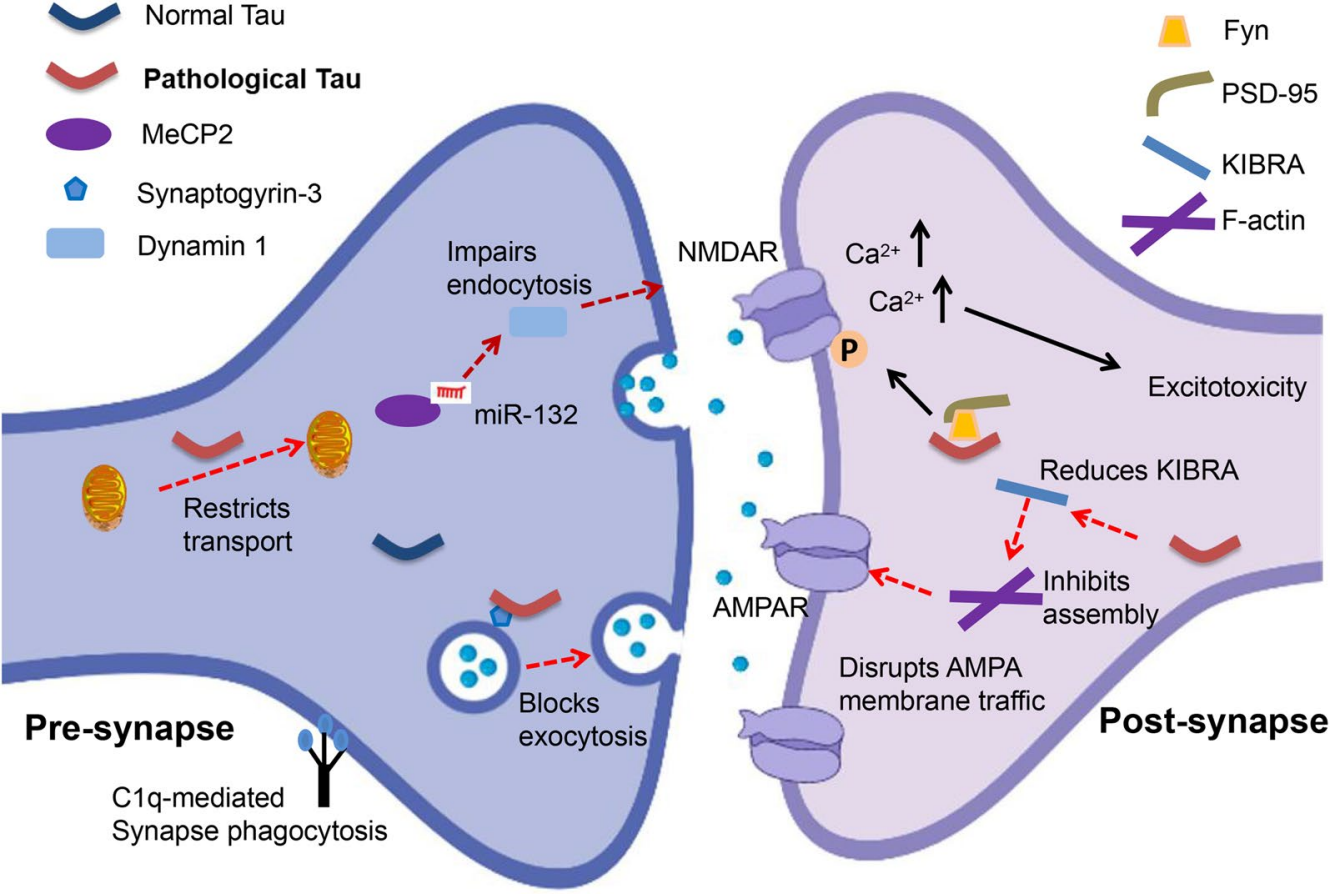
Pathological tau induces synaptic dysfunction. At presynaptic terminals, the interaction of pathological tau with synaptogyrin-3 on synaptic vesicles hampers vesicle mobility and impairs presynaptic vesicle cycling. Pathological tau impairs neuronal endocytosis through the miR-132–meCP2–dynamin 1 pathway. Pathological tau induces tagging of synapses by complement initiation factor (C1q) and activates synapse phagocytosis by microglia. The infiltration of pathological tau into postsynapses recruits Fyn to NMDAR/PSD-95 complexes and causes excitotoxicity mediated by amyloid-β and excessive glutamate. Accumulation of acetylated tau contributes to KIBRA deficiency, which blocks the activity-dependent F-actin polymerization and disrupts AMPA receptor membrane anchoring at postsynapses

The presence of pathological tau in the presynaptic compartment is linked to the decreased mobility and release rate of synaptic vesicles [35] and the pathogenesis of AD [68]. Tau interacts with a subset of presynaptic proteins, including synaptophysin, synapsin-1, synaptotagmin, synaptogyrin-3, syntaxin-1B, α-synuclein and β-synuclein [69]. Microinjection of recombinant human tau into the presynaptic terminals results in transient neurotransmitter release mediated by calcium, persistent synaptic depression failure, and disruption of synaptic transmission, when using a squid giant synapse model [70]. McInnes et al. [71] have found that tau with N-terminal sequence can bind to synaptogyrin-3, a presynaptic vesicle-associated protein, causing excessive aggregation of synaptic vesicles and restricting presynaptic vesicle mobility, thereby attenuating neurotransmission in fly and mouse models of tauopathy. On the other hand, reducing synaptogyrin-3 expression prevents tau from binding to vesicles, attenuates the vesicle mobility defects, and restores neurotransmitter release, thereby alleviating early synaptic dysfunction in neurodegenerative diseases [71] (Fig. 1). Moreover, Largo-Barrientos et al. [72] have shown that tau binds to synaptogyrin-3 on synaptic vesicles and excessively aggregates at presynaptic terminals, driving synaptic dysfunction in PS19 mice. The presynaptic pathological tau directly induces working memory defects, which are dependent on the presence of synaptogyrin-3. Synaptic loss and memory decline can be alleviated by lowering synaptogrin-3 expression [72]. The pathological tau also causes neuroinflammation, which is involved in axonal trafficking impairment, membrane disruption, and reactive oxygen species production [73, 74]. The presence of pro-aggregated tau at presynapses contributes to the terminal enlargement and synaptic vesicle pool exhaustion, along with impaired synaptic plasticity [75]. The pathological tau impairs presynaptic function in the entorhinal cortex in models of early AD [66]. It also impairs neuronal endocytosis through the miR-132–methyl-CpG binding protein 2 (MeCP2)–dynamin 1 pathway, which represents a possible mechanism underlying the role of tau in presynaptic dysfunction [76] (Fig. 1). Further studies are needed to address whether direct regulation of this pathway in vivo can ameliorate the presynaptic disorder caused by pathological tau in AD. In conclusion, these data suggest an essential role for pathological tau in presynaptic dysfunction. However, the precise mechanisms through which pathological tau in presynaptic compartments induces synaptic dysfunction and cognitive decline remain to be investigated.

### Pathological tau induces postsynaptic dysfunction

Growing evidence implicates that the infiltration of pathological tau to the postsynapses contributes to the postsynaptic excitotoxicity and memory deficits by impairing the trafficking of NMDARs and AMPARs and synaptic anchoring in dendritic spines and postsynapses [11, 77–79]. Tau at postsynaptic terminals can promote the interaction between GluA2 subunit of AMPAR and PICK1 (protein interacting with C kinase 1), supporting the critical role of tau in regulating AMPAR endocytosis and hippocampal LTD in vitro [80]. In postsynaptic compartments in vivo, tau interacts with the PSD-95/NMDAR complexes through binding with the Src tyrosine kinase Fyn, a substrate of which is NMDAR [34, 81]. Under pathological conditions, missorted tau acts as a scaffold protein to deliver more Fyn to postsynaptic compartments and promotes phosphorylation of the NR2B subunit of NMDARs [34]. This induces the excessive activation of NMDARs, Ca^2+^ influx, and neuronal excitotoxicity [16, 34] (Fig. 1). Saracatinib (AZD0530), a Fyn inhibitor, was able to rescue the synaptic density loss and synaptic dysfunction in a mouse model of AD [82]. In a Phase Ib clinical trial (NCT01864655) in patients with mild-to-moderate AD, AZD0530 could achieve substantial CNS penetration through oral dosing at 100–125 mg [83], and later in a multicenter Phase IIa randomized clinical trial (NCT02167256), it showed a trend for slowing the reduction of hippocampal volume and entorhinal thickness [84]. Furthermore, expression of truncated tau or tau deficiency prevents Fyn from trafficking into postsynaptic terminals, thus improving the survival rate of the APP23 AD mouse model [34, 85]. Tau is a substrate of GSK3β and p38 mitogen-activated protein kinase (p38MAPK) which exist in postsynaptic compartments and participate in the regulation of synaptic function, especially LTP [2, 86, 87]. Tan et al. [88] revealed that isoorientin, a GSK-3β inhibitor, attenuates phosphorylation of tau and rescues synaptic dysfunction in APP/PS1 model mice. Tideglusib, another competitive GSK-3β inhibitor, showed good tolerance and safety, and improved the cognitive function of patients with mild-to-moderate AD at a specific concentration in a Phase II clinical trial, but did not produce clinical efficacy [89]. Tau phosphorylation and acetylation play an essential role in tau mislocalization and postsynaptic dysfunction. Abnormally phosphorylated tau reduces the trafficking of glutamate receptor subunits GluA1 and GluA2/3 to PSD-95 [11, 90]. In addition, a mimic of phosphorylated tau impairs postsynaptic function through global disruption of synaptic trafficking or anchoring of AMPARs and NMDARs in cultured mouse neurons [11]. Tau is also required for the formation of dendritic spines and PSD, and tau deficiency or mutation impairs the sensitivity of newborn granule neurons to modulation of hippocampal neurogenesis and synapse maturation [91, 92]. Elimination of tau-specific phosphorylation at KxGS motifs by AMP-activated kinase counteracts the loss of dendritic spines and restores the synaptic function of hippocampal neurons both in vitro and in APP mouse models [93]. On the other hand, p38γ-mediated phosphorylation of tau inhibits the Aβ-induced excitotoxicity in both cellular and APP23 AD mouse models, suggesting a protective role of phosphorylated tau in postsynaptic terminals [94]. In addition, accumulation of K281- and K274-acetylated tau causes hippocampal LTP impairment and memory deficits, which are attributed to the reduction of memory-associated postsynaptic Kidney/BRAin (KIBRA) protein, as well as postsynaptic F-actin remodeling and disruption of AMPAR membrane anchoring, in both AD patient brains and transgenic mice expressing human tau that mimics K281 and K274 acetylation [12] (Fig. 1). Min et al*.* have demonstrated that salsalate, a drug that inhibits the activity of p300 to deacetylate tau, can potentially be used to improve synaptic dysfunction and cognitive decline [95]. Enhancing KIBRA levels or restoring the downstream signal transmission at postsynapses may be another strategy for AD therapeutic intervention [96]. In addition, miR-135a-5p, which is reduced in excitatory hippocampal neurons in a tau-dependent manner in AD mice, mediates the cytoskeleton re-organization disorder and synaptic impairments in these excitatory hippocampal neurons [97]. miRNAs are the most important post-transcriptional regulators, acting as fine-tuners for synaptic plasticity, and how they interact with pathological tau to induce postsynaptic dysfunction needs to be further investigated.

### Extracellular pathological tau contributes to synaptic dysfunction

In addition to regulating intracellular synaptic transmission, tau also has extracellular functions. Four different manners of tau release have been proposed, including direct translocation from the cytoplasm across the plasma membrane, lysosomal release, release via secretory exosomes, and micro-vesicle shedding [13]. The direct translocation across the membrane to extracellular space is an unconventional pathway of tau secretion. The secretion of tau increases with the degree of its phosphorylation, and sulfated proteoglycans on the plasma membrane facilitate its export [98]. Potassium-induced depolarization demonstrates increased release of tau and tau fragments from the presynaptic terminals in AD postmortem samples compared to control samples [99]. The extracellular tau regulates the signal transduction of synaptic receptors, such as muscarinic acetylcholine receptors (mAChRs) [2, 100]. The binding affinity of extracellular recombinant tau to mAChRs is much higher than that of acetylcholine, thus affecting the interneuronal signal transmission [101, 102]. It has been reported that extracellular oligomeric tau impairs LTP memory in mice [103, 104]. Neuron treatment with oligomeric tau impairs the morphology and density of dendritic spines, along with an increase in the concentration of intracellular reactive oxygen species and calcium [105]. In addition, extracellular oligomeric tau absorbed by neurons triggers abnormal tau accumulation, impairs the rapid transport of axons, and disrupts normal neuronal homeostasis [106]. Injection of N-terminal fragments of tau induces alterations of synaptic activity independently of overt neurodegeneration [107]. Furthermore, extracellular tau leads to synaptic dysfunction when secretomes from the human induced pluripotent stem cell-derived neuronal AD models are injected into the rat brain [108]. Application of a monoclonal antibody that recognizes and blocks extracellular tau aggregates can reduce the activation of microglia and improve cognitive deficits in P301S transgenic mice [109]. Another monoclonal antibody, DC8E8, can inhibit extracellular tau–tau interactions, discriminate between healthy and pathological tau, reduce tau pathology in mouse tauopathy models, and inhibit internalization of extracellular pathogenic tau in AD neurons in vitro [110]. Nevertheless, the underlying mechanisms of synaptic dysfunction caused by extracellular pathological tau need to be further investigated. Identifying and characterizing elements that promote or participate in the restriction and degradation of extracellular pathological tau will help to develop novel therapeutic approaches for AD treatment.

## Pathological tau and mitochondrial dysfunction at synapses

The synaptic terminal is an energy high-demanding place, and the process of synaptic transmission requires high levels of ATP for exocytosis and release of neurotransmitters [111]. Mitochondrial dysfunction plays a key role in the occurrence and development of AD [112]. When mitochondria cannot be efficiently transported into synapses, ATP production and calcium buffering would be diminished, and the trafficking of glutamate receptor subunits to the postsynaptic membrane is impaired, ultimately resulting in synaptic dysfunction [113]. Pathological tau plays a vital role in impairing mitochondrial transport, thereby reducing the number of presynaptic mitochondria and hampering the release of synaptic vesicles [35, 114, 115]. In cultured neurons from P301L tau knock-in mice, the number of mitochondria is significantly decreased in axons and the volume of individual motile mitochondria increased, indicating that tau regulates mitochondrial functions [116]. In addition, the interaction between phosphorylated tau and dynamin-related protein 1 (Drp1), a mitochondrial fission protein, enhances excessive mitochondrial fragmentation, causing mitochondrial and synaptic degeneration, ultimately leading to neuronal damage and cognitive decline in AD [117]. Furthermore, tau and GSK-3β double-transgenic mice exhibit elevated Drp1 expression and mitochondrial fragmentation [118]. Treatment designed to reduce Drp1 expression can decrease the interaction between Drp1 and phosphorylated tau, thereby blocking the mitochondrial abnormalities and synaptic damage in AD [119]. The synaptic protein α1-takusan directly or indirectly interacts with tau and PSD-95 to prevent the Aβ-induced tau hyper-phosphorylation and mitochondrial fragmentation at postsynaptic terminals, thereby protecting the synapse [120]. Mitochondria in AD animal models and brains of AD patients are enriched in N-terminal tau fragment (tau26-230), which is associated with mitochondrial and synaptic dysfunction [121]. A recent study reported that the presence of truncated tau at Asp421 impairs mitochondrial dynamics by reducing the level of optic atrophy protein 1 (Opa1) in AD [122]. The relationship between pathological tau and Opa1 expression, and the relationship between downregulation of Opa1 and mitochondrial integrity in AD need further clarification. In addition, approaches to reducing abnormal interactions between pathological tau and mitochondrial proteins to increase the number and quality of mitochondria in synapses are worthy of investigation.

## Effects of microglia in tau-induced synaptic dysfunction

AD is accompanied by neuroinflammation, manifested by microgliosis, astrogliosis, and elevated pro-inflammatory cytokine levels. Microglia are macrophages residing in the brain, which originate from erythromyeloid progenitor cells derived from the yolk sac [123]. Microglia can respond to inhibitory and excitatory neurotransmitters, and interact with neuronal synapses in an activity-dependent manner [124]. They can also engulf the tau-positive synaptic compartments of neurons with aberrant pathology via a complement-dependent mechanism, leading to synaptic dysfunction [125]. In particular, the classical complement initiator C1q is upregulated in patients with AD and tauopathy and has been detected to be present with hyperphosphorylated tau in synapses of AD patients and PS19 mice, which is associated with microglial phagocytosis of synapses and synaptic density decline [125, 126]. The tau-affected and C1q-tagged synapses are further tagged with the central complement component C3, and engulfed and phagocytosed by microglia [126, 127]. Moreover, the inactivation of complement C3a receptor which predominantly originates from microglia, can attenuate tau pathology and rescue synaptic deficits and neurodegeneration in the PS19 mouse model of tauopathy [128]. C1q and C3 are located at synapses and trigger microglia to engulf the synapses. Antibodies against C1q have been shown to reduce the pathological tau-mediated synaptic engulfment by microglia [126]. Gratuze et al. [129] have hypothesized that synaptogyrin-3 on synaptic vesicles clusters at the presynaptic terminal through interaction with phosphorylated tau, leading to synaptic tagging with C1q and downstream synaptic deposition of C3, and finally synapses are engulfed by microglia [129] (Fig. 1). More studies are needed to uncover the association between pathological tau and C1q-mediated tagging of synapses to protect synapses from microglial phagocytosis. In addition, fractalkine, a unique chemokine that suppresses the expression of pro-inflammatory genes, can reduce tau hyper-phosphorylation and neurodegeneration, and improves cognitive deficits by inhibiting the activation of microglia in a tauopathy mouse model [130].

## Conclusions and perspectives

**Fig. 2:**
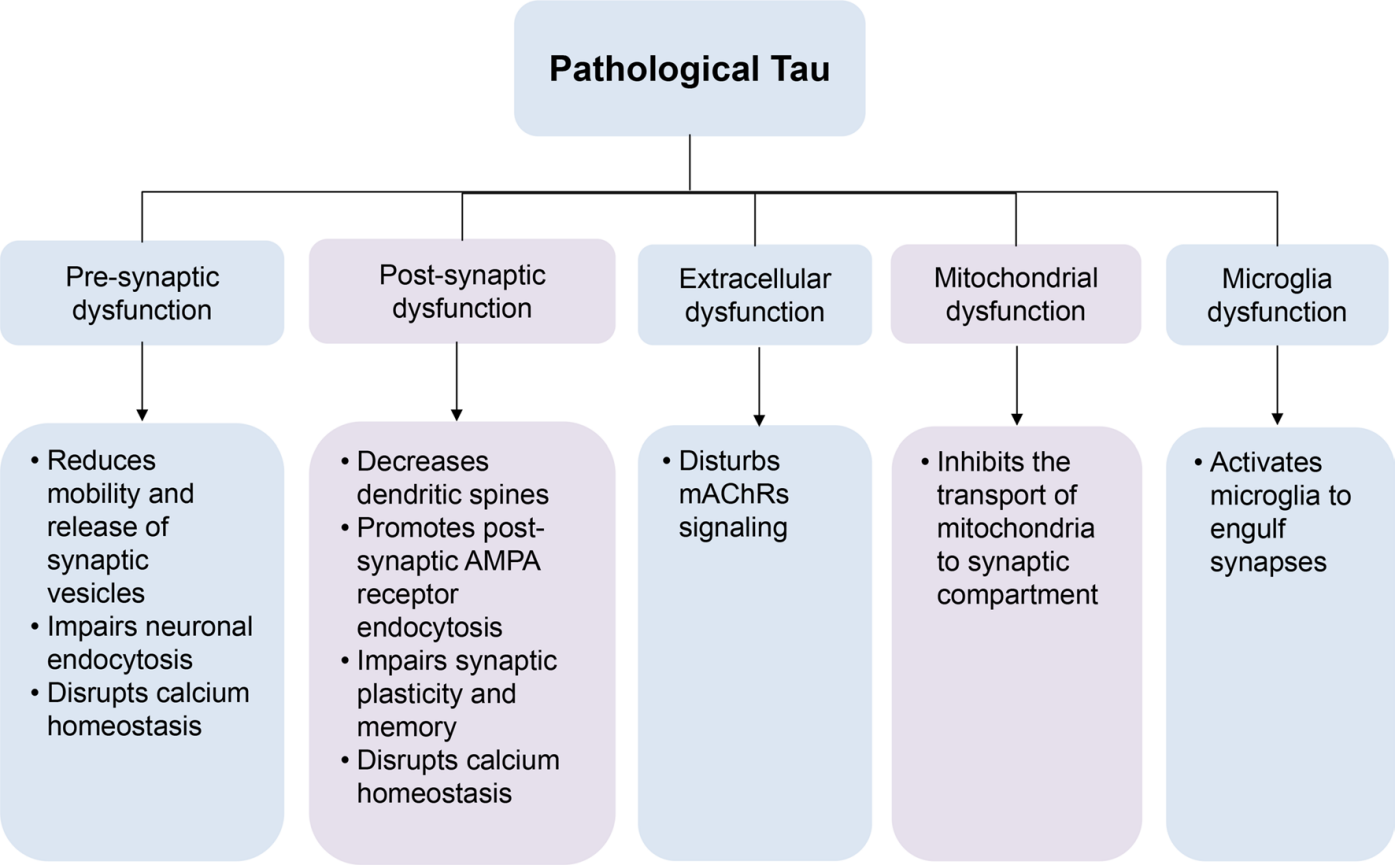
Pathological tau promotes synaptic dysfunction by multiple mechanisms in early stages of Alzheimer’s disease

Synaptic dysfunction occurs in early AD and is closely associated with cognitive decline. Tau plays an important role in altering synaptic function during the pathogenesis of AD. Recent advances reviewed here shape our understanding of how pathological tau contributes to synaptic dysfunction. The pathological tau clearly promotes synaptic dysfunction through multiple mechanisms, including affecting the regulation of dendritic spine density, the presynaptic vesicle dynamics, the engulfment of synapses, the synaptic mitochondrial transport, and the synaptic plasticity (Fig. 2). It is crucial to determine the impact of pathological tau on synaptic loss and cognitive impairment, especially since there is no effective treatment for AD. Targeting synapses may also be an attractive therapeutic strategy. Therefore, the relationship between pathological tau and synaptic dysfunction should be further studied to understand its toxic effects, and to provide insights into new therapeutic targets for maintaining synaptic number, structure and function and slowing cognitive decline in AD.

## Data Availability

Not applicable.

## Abbreviations

AD: Alzheimer’s disease
Aβ: Amyloid-beta
NFTs: Neurofibrillary tangles
MTR: Microtubule-binding repeat
LTP: Long-term potentiation
LTD: Long-term depression
GSK-3β: Glycogen synthase kinase 3
AMPAR: α-Amino-3-hydroxy-5-methyl-4-isoxazolepropionic acid receptor
NMDAR: N-methyl-D- aspartic acid receptor
PSD: Postsynaptic density
mAChRs: Muscarinic acetylcholine receptors
KIBRA: Memory-associated postsynaptic Kidney/BRAin
Drp1: Dynamin-related protein 1
